# Disentangling the role of parasite infectivity and density from host susceptibility in infection development and parasite proliferation

**DOI:** 10.1098/rsbl.2025.0356

**Published:** 2025-11-26

**Authors:** Christina Pernice Tadiri, Dieter Ebert

**Affiliations:** ^1^ Department of Environmental Sciences, Zoology, University of Basel, Basel, Basel-Stadt, Switzerland

**Keywords:** *Daphnia magna*, *Pasteuria ramosa*, disease ecology, host–parasite coevolution

## Abstract

Understanding the environmental drivers of host–parasite interactions is a major concern to human health and conservation, particularly in the context of emerging infectious diseases. The likelihood of contracting an infection can be related to both the rate of contact between host and parasite, as well as innate features of hosts (susceptibility/resistance) and parasites (infectivity, virulence, within-host proliferation rate). This study uses a host–parasite system with a matching-allele model for host susceptibility and parasite infectivity to disentangle contact rate from parasite infectivity while accounting for the effects of host susceptibility. Using three exposure doses from several parasite isolates to hosts with known susceptibility, we find significant differences in parasite infectivity (in terms of number of successful infections) and within-host proliferation rate among parasite isolates, after controlling for exposure rate and host genotype. Host known susceptibility also had a strong impact on parasite infection success and proliferation rate, due to the nature of this host–parasite system. The exposure dose did not impact the number of infections or infection intensity. No significant relationship between infection success and parasite proliferation rate was detected, indicating a weak or non-existent covariance among isolates for both variables.

## 1. Introduction

Infectious diseases are of important concern to human health and species conservation [1], exemplified by the rise of emerging epidemics globally over the past decades [1,2]. Host–pathogen interactions are often governed by feedback loops in which the parasite population grows and spreads in response to host population dynamics and demography. This, in turn, regulates host population dynamics, leading to epidemics in which the effects of individual variables are difficult to isolate. Longer term, parasites and hosts reciprocally select for various traits, and this selection may also influence overall community dynamics [3]. The predictions of more emerging pandemics in the future [1,4] demonstrate that now, more than ever, it is important to further our understanding of infectious disease epidemiology and transmission dynamics, particularly in terms of factors governing transmission success and parasite virulence. Disentangling environmental and genetic parasite traits and their reciprocal action is critical in this endeavour.

To predict host–parasite interactions, it is crucial to understand the interplay between transmission (parasite encounter/exposure rate and parasite infectivity), parasite virulence and host susceptibility. Transmission is arguably the most important determinant of epidemic dynamics, yet in mathematical models, it is often reduced to a single term [5]. A major factor governing transmission is the rate of contact between parasites and hosts, which is often dependent on parasite density in a host population. Beyond encounter rates, parasite infectivity and host susceptibility are two important variables that determine transmission success [5]. Considerable heterogeneity may exist in these two characteristics, and they are the main drivers of host–parasite coevolution. Parasite virulence is typically defined as the severity of a parasite’s impact on host health, although for plant hosts, it is defined simply as the ability to cause disease. The severity of impact on a host’s health is often considered to be related to the parasite proliferation rate inside the host, which may also impact transmission stage production and thus future transmission opportunities and successes by increasing exposure probability. Here, we therefore specifically use parasite proliferation (i.e. transmission stage production) as a proxy for virulence. It has long been hypothesized that trade-offs between transmission stage production (and thus transmission success) and virulence exist for parasites [6], where increasing virulence may reduce transmission opportunities through increasing host mortality. However, slower reproduction within a host also reduces opportunities for transmission; therefore, intermediate levels of virulence are expected to result in the greatest transmission [7]. When transmission opportunities are high, for example, at high encounter rates, higher virulence may also be favoured [8]. Considerable research has been dedicated to the transmission–virulence trade-off, and a more recent meta-analysis of 29 studies across a range of host–parasite systems generally supported the idea of a positive relationship between parasite replication and virulence as well as parasite replication and transmission [9]. However, it was unable to determine a significant relationship between virulence and transmission and noted that further studies are needed and that disentangling virulence from transmission is often logistically difficult. It is also unclear whether this trade-off would still exist for parasites with a persistent environmental stage (where transmission is less ‘urgent’) or for those that obligatorily kill their host to transmit. The aim of this experiment was to disentangle parasite infectivity and virulence (using proliferation rate as a proxy) from environmental density while accounting for host specificity using a well-studied model system involving water flea (*Daphnia magna*) hosts and their environmentally transmitted bacterial parasite *Pasteuria ramosa* to determine the relationship between environmental density, infectivity and virulence.

*Daphnia* spp. are small aquatic crustaceans broadly distributed on all continents except Antarctica [10,11] and are one of the most studied organisms worldwide. They are often used as model organisms in ecology, evolution and epidemiology [12]. *Pasteuria ramosa* is a bacterial endoparasite of *D. magna*, with a strong impact on host fitness and population dynamics [13–15]. The parasite transmits horizontally from environmental reservoirs. The transmission stages (spores) of the parasite are long lasting and overwinter in the pond sediments [16]. *Pasteuria ramosa* spores are ingested by *Daphnia* when filter feeding. During parasite proliferation inside the host, hosts lose the ability to reproduce (parasitic castration), exhibit gigantism and become a bright orange or dark red, allowing one to determine whether an individual is infected without destructive sampling. This parasite obligatorily kills its host, at which point spores are distributed back into the sediment when the host body decays. This host and parasite are a well-known model system for coevolution [17,18]. Importantly, a matching-allele-infection matrix has been described for this host and parasite such that host susceptibility and parasite infectivity are highly genotype-specific [19], resulting in various host ‘resistotypes’ (innate resistance to a diverse panel of parasite isolates). Previous work has also suggested that intermediate virulence (in terms of time to host mortality) maximizes spore production within the host [20].

Recent work has demonstrated that certain ‘resistotypes’ of *D. magna* are less likely to become infected and take longer to show signs of visible infection when exposed to both pond sediments (which contain parasite transmission stages) and water from a natural population in Lake Aegelsee in Switzerland containing a diversity of *P. ramosa* spores [21]. However, it is unclear whether this is due to a lower frequency in the environment of spores to which they are susceptible and therefore lower exposure rates, or if the parasite isolates capable of infecting these hosts are generally less successful at transmitting, or slower to reproduce even within susceptible hosts, resulting in later symptoms (i.e. less virulent). Previous work has shown some variability in parasite proliferation rate among some *P. ramosa* isolates [22]; however, that study only used one host genotype and one spore concentration for all exposures; thus, it remains unclear whether differences in parasite reproduction solely explain the differences in prevalence observed in nature, or if host susceptibility and parasite environmental density also play a role in epidemic dynamics. Another study using a broad range of parasite doses but only one parasite isolate and one host clone found that parasite within-host proliferation peaked around 1000 spores [23] with lower and higher doses resulting in lower spore production. Further studies using more host–parasite combinations and a broader range of doses found that dose had a positive impact on infection success within certain host–parasite combinations [24,25]. However, they did not determine whether within-host spore production was impacted by initial dose, host clone or parasite isolate.

Thus far, studies have not specifically disentangled parasite proliferation rate (as a proxy for virulence) from host susceptibility or parasite infectivity and initial parasite exposure/dose. Understanding this dynamic is important to making predictions about epidemic dynamics and host–parasite coevolution. If transmission is density-dependent, it would imply that infections are simply occurring as hosts encounter parasites to which they are susceptible and that the relative concentrations of these various parasite isolates are maintained in a natural system at nearly constant proportions year after year. If the observed differences are due to biological differences in virulence among parasite isolates, it could imply that selection for lower parasite virulence is occurring alongside selection for host resistance. We present an experiment conducted under controlled laboratory conditions to determine which of these possibilities are more likely.

## 2. Methods

### (a) Experimental design

We aimed to determine whether some parasite isolates are more infectious and/or more productive than other isolates even when presented with ‘susceptible’ hosts, while controlling for their potentially lower densities in a natural setting which would decrease the probability of contact with a susceptible host. Six different parasite isolates, falling within three major evolutionary lineages [26], were used at three different ‘doses’ (densities) to expose nine different host genotypes in a full-factorial design.

Parasites from laboratory-propagated isolates were used for this study. Four *P. ramosa* C1, C19, P15 and P21 were previously described [27] and were obtained from infected individuals in Russia, Germany and Belgium, representing two distinct parasite lineages. Two newly acquired isolates obtained from Lake Aegelsee in Switzerland, P38 and P54 were also used, representing a third lineage [26]. Nine host clones were selected from a laboratory panel of *D. magna* obtained from all over the Holarctic. They were selected based on their previously determined resistance or susceptibility to these parasite isolates based on attachment tests such that a broad spectrum of susceptibility to all parasite isolates was covered [28]. Selected host clones came from Denmark, Germany, Russia and Switzerland and were chosen if they were either uniquely susceptible to only one of the three parasite lineages, or to all three parasite lineages, in order to control for the known gene × gene interactions in this system.

A total of 10 replicates of the full experimental design (six parasite isolates × three doses × nine host clones × 10 replicates = 1620 individuals) was performed.

### (b) Experimental exposures and sampling

Parasite spores were propagated following a standard procedure, starting from frozen infected host individuals infected with a single, known isolate from our parasite laboratory panel. Hosts were crushed to release spores, shaken for 2 h at 4250 rpm to separate spores, and the concentration of spores was counted using a haemocytometer. Spore suspensions were produced to achieve the desired number of spores. *D. magna* clonal lines were cultured in 380 ml jars of ADaM (media) [29] in incubators at the same environmental conditions as the experiment to avoid variation in maternal effects (20°C, 16 h light : 8 h dark cycle and 80% humidity and fed with the green algae *Tetradesmus obliquus*).

Juvenile *D. magna* were isolated in 100 ml jars with 50 ml of *Daphnia* medium (ADaM) and infected with 10 000 spores, 20 000 spores and 40 000 spores. These three doses are lower than the standard dose used to infect *D. magna* in the laboratory (80,000). Our aim was not to reach high infection rates, but to see differences in infection rates among host and parasite combinations while controlling for exposure rate by using known concentrations, especially at low concentrations like those that may be encountered in the wild. Individuals were monitored for signs of visible infection (gigantism, no eggs in the brood chamber and red/orange body coloration) three times weekly for four weeks. After 28 days, all individuals showing any of the potential signs of infection were preserved in 1 ml of media in an Eppendorf tube and frozen, as previous work has shown that all infected individuals experience parasitic castration within one month [13]. Individuals were later defrosted and crushed, shaken at 4250 rpm for 2 h, and then spores were counted twice using a haemocytometer to determine the number of spores per millilitre. These data allow the calculation of the total number of spores produced by an individual infection.

### (c) Statistical analysis

All statistical analyses were performed in R [30]. Significance level was set to *p* < 0.05. Reported values are means ± standard error unless otherwise stated. Raw data and its description are available at [31].

### (d) Parasite infectivity

Parasite infectivity was defined as infection success in an individual host, measured by whether any signs of infection were displayed within 28 days and confirmed by the presence of parasite spores when crushed and inspected at 200× magnification. Infectivity was compared among parasite isolates using logistic regression with host genotype, parasite isolate and dose (as a factor due to potential nonlinearity) as independent variables. Although gene-for-gene interactions are known to determine whether a specific parasite isolate can infect a specific host clone, our aim was to assess variability among parasite isolates in their infectivity while controlling for the effects of host susceptibility. Thus, we designed our experiment to include a range of matched and mismatched host–parasite combinations, and we did not include an interaction term in our model but left both host and parasite as main effects. Model fit was assessed using a likelihood ratio test compared to a null model. *Post hoc* Tukey tests were performed to determine the differences among pairs of parasite isolates if significant in the full model.

### (e) Parasite proliferation

Parasite proliferation was estimated only in individual hosts that became infected as total spore production over the 28 days, based on the average number of spores per millilitre based on our two haemocytometer counts. Parasite proliferation (total spores produced) was log-transformed and compared among parasite isolates using a generalized linear model. Again, host genotype, parasite isolate and dose (as a factor) were used as independent variables. As only hosts which became infected were included in this analysis, we remove any gene × gene interaction associated with infection success that has already been documented in this host–parasite system. Model fit was assessed using a likelihood ratio test compared to a null model. *Post hoc* Tukey tests were performed to determine the differences among pairs of parasite isolates if significant in the full model.

### (f) Relationship between proliferation and infectivity

We tested for any relationship between infection success and parasite proliferation using a Spearman correlation performed for the average infectivity and proliferation of each parasite isolate across all host clones and doses.

## 3. Results

A total of 1620 individual *D. magna* were exposed to an array of parasite isolates and doses, of which 217 became infected within 28 days. This relatively low number of infections was expected, as we included low spore doses and because many of the combinations of parasites and hosts within the design were with host clones believed to be resistant to many of the specific parasite isolates used. ANOVA tables of models and outputs of Tukey pair-wise comparisons for all significant factors can be found in electronic supplementary materials.

**Figure 1.**
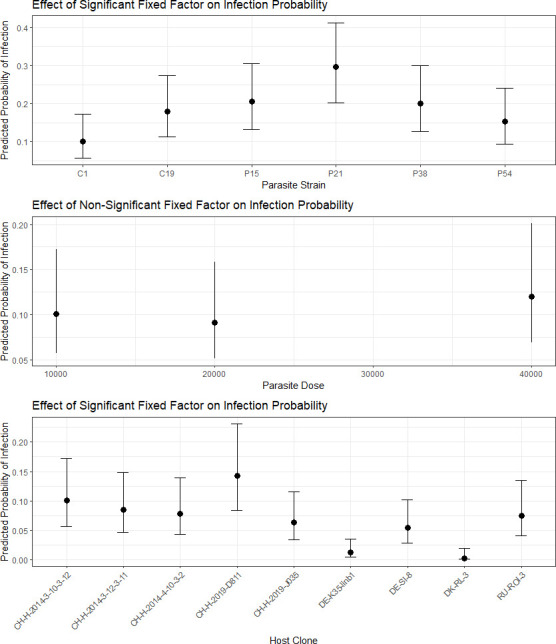
Predicted impact of parasite isolate, parasite dose and host clone effects on probability of infection based on our multivariate logistic regression. Points represent the estimated probabilities, and error bars indicate the 95% confidence intervals. Infection success was significantly influenced by both parasite isolate and host clone, but not dose.

Our model for infection success (figure 1) included dose, parasite isolate and host clone and significantly improved upon the null model (X^2^ = 126.6, d.f. = 15, *p* < 0.001). Host clone had a significant impact on whether an individual became infected (X^2^ = 98.5, d.f. = 8, *p* < 0.001). Parasite infection success was also significantly affected by parasite isolate (X^2^ = 26.8, d.f. = 5, *p* < 0.001), and *post hoc* analysis revealed significant differences in infection success between isolate P21 and C1 (estimate = 1.33 ± 0.29; *p* < 0.001) and between isolate P21 and P54 (estimate = 0.85 ± 0.25; *p* = 0.01). Overall, infection success ranged from 7.1% (C1) to 21.1% (P21), with the other four isolates all infecting around 13% of individuals they were exposed to. The effect of initial spore dose on infection success was not significant (X^2^ = 2.87, d.f. = 2, *p* = 0.24).

**Figure 2.**
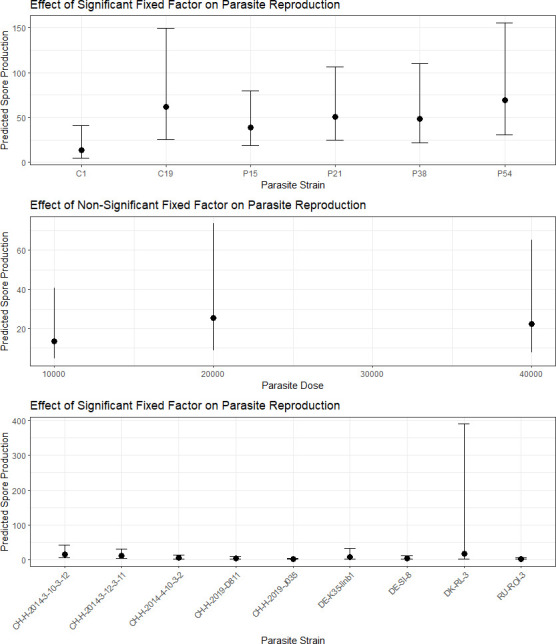
Predicted impact of parasite isolate, parasite dose and host clone effects on parasite proliferation based on our multivariate logistic regression. Points represent the estimated probabilities, and error bars indicate the 95% confidence intervals. Proliferation was significantly influenced by both parasite isolate and host clone, but not dose.

Our model for parasite proliferation (figure 2) included dose, host clone and parasite isolate and significantly improved upon the null model (X^2^ = 201.54, d.f. = 15, *p* < 0.001). Host clone had a significant impact on within-host parasite proliferation among individuals that became infected (X^2^ = 57.04, d.f. = 8, *p* < 0.001). Parasite isolate also had a significant impact on within-host parasite proliferation (X^2^ = 12.69, d.f. = 5, *p* = 0.03). The *post hoc* Tukey test revealed significant differences in spore production between isolates C19 (estimate = 1.5 ± 0.47, *p* = 0.02) and P54 (estimate = 1.62 ± 0.53, *p* = 0.03) with isolate C1. Overall, the average number of spores produced within 28 days in infected individuals ranged from 7.53 × 10^4^ ( ± 3.33 × 10^4^) for isolate C1 to 9.74 × 10^5^ ( ± 2.97 × 10^5^) for isolate P38. No significant effect of dose was detected (X^2^ = 5.62, d.f. = 2, *p* = 0.06).

**Figure 3.**
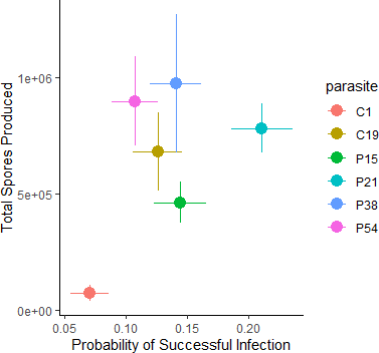
Parasite infectivity (probability of successful infection) and virulence (using parasite proliferation within infected hosts as a proxy) among parasite lineages. Parasite proliferation was measured as the total spores produced in an individual over 28 days.

Overall, no significant or strong relationship between infection success and parasite reproduction was observed among isolates (Spearman correlation: rho = 0.26, *p* = 0.67; figure 3). Parasite isolates ranged from having both the lowest infection success rate and producing the fewest spores within individuals that they did infect (C1), to having relatively high infection success and producing an intermediate number of spores (P21).

## 4. Discussion

This study aimed to disentangle differences in within-host proliferation (a proxy for virulence) and infectivity among parasite isolates from differences in host susceptibility and infection dose (parasite density) to determine whether differences in the time to visible symptoms observed among hosts exposed to wild parasite populations [32] are due to lower concentrations of certain parasite isolates or suitable hosts, or differences in proliferation or infectivity. We found, after accounting for host differences in susceptibility, significant differences in parasite infection success and proliferation in infected hosts, indicating differences in both infectivity and virulence among parasite isolates, independent of the density of parasite spores to which the host was exposed. We found no correlation between infection success and parasite reproduction, indicating that these two metrics of virulence may not necessarily trade off in this system.

Unsurprisingly, host genotype accounted for a large amount of the variance for both infection success and parasite reproduction. Given that host susceptibility and parasite infectivity in this system are known to show extreme genetic variation and to follow a matching-allele model [19], it was expected that the majority of combinations in this experimental design would not result in infections due to mismatches between host and parasite and that host genotypes would have an important impact on infection success and parasite proliferation. We control for these effects of host susceptibility in two ways to isolate the variables of parasite infectivity and parasite proliferation among isolates: first, by using an array of host genotypes with unique susceptibility profiles (including some that were susceptible to all parasites) and second, by including it in our multivariate models. In doing so, we are able to disentangle parasite genotype from environmental density while accounting for the fact that this host–parasite system is genotype-specific. We still find a profound effect of parasite isolate on both infection success and parasite reproduction across all our host clones when the effects of host clone are held constant. These findings are similar to earlier work which found variability in parasite proliferation rate among isolates when exposed to a single, broadly susceptible host clone [22], but strengthen the conclusions by using an array of host clones.

We control for any effects of different exposure rates due to different isolate densities in nature by using the same number of spores for each parasite isolate, replicated at three different concentrations. Previous work had found a positive effect of dose on infection success [23–25], using doses much higher than those in our study. We were unable to replicate these results for infection success or parasite proliferation, possibly due to the much lower spore concentrations and a smaller range among doses used here. This study adds to the literature by specifically exploring differences in parasite proliferation among parasite isolates while controlling for host susceptibility and initial exposure dose. It also indicates that differences in infection levels observed when using a sample of parasite populations from nature [21] may be due to differences in proliferation rate among different parasite isolates. Detecting spore concentrations in nature that typically result in successful infection would be an important avenue for future research. Nevertheless, we show that independent of initial parasite exposure, parasite isolates vary significantly in both their infectivity and within-host proliferation when exposed to susceptible hosts.

Interestingly, we found neither a positive nor negative relationship between infectivity and parasite proliferation within infected hosts. A positive relationship would have implied that poor infectors are also poor at reproducing while successful infectors also reproduce the most, indicating that both metrics are somehow associated with parasite fitness and consistent with a meta-analysis which found a generally positive, decelerating relationship between transmission and virulence [9]. In this study, we saw that the isolate least capable of infecting (C1) also produced the fewest spores in individuals it did infect. However, the one that was best at infecting (P21) had a higher proliferation rate than C1, but not significantly higher than any other strains, whereas those with the highest spore production (P38 and P54) had an intermediate/low infection rate. Theory often predicts that evolutionary trade-offs between parasite within-host proliferation and infectivity may exist [9]; however, this trade-off is due to the probability of opportunity to infect the next host, which would not be relevant for *P. ramosa* since it obligatorily kills its host to transmit and produces a long-lasting environmental stage. Our results imply that infectivity and proliferation of this parasite are not necessarily related to each other, though given our relatively small sample size, it is not possible to completely rule out a relationship between infectivity and proliferation for this parasite. Overall, our results highlight the variability in both infectivity and proliferation, even among parasites of the same, highly host-specific parasite species.

Through controlled factorial laboratory experiments, we demonstrate distinct differences in parasite within-host proliferation and infectivity within a single host–parasite system, even after accounting for variability in host susceptibility and encounter rate. We also show that within a single parasite species, a wide variability in both infectivity and virulence exists, with no discernable relationship between these two variables. These findings also imply that it may be difficult to predict parasite virulence in real-world settings, where several factors including host density and susceptibility, parasite frequency and numerous environmental variables that may impact host–parasite interactions are at play. Deepening our understanding of how hosts and parasites respond long term not only to each other but to the broader environment would be an important avenue for future work, particularly in the face of changing environments.

## Data Availability

All raw data and a data description are available from the Dryad Digital Repository [31]. Supplementary material is available online [33].
